# Influence of Dental Restorations on Oxidative Stress in Gingival Crevicular Fluid

**DOI:** 10.1155/2018/1823189

**Published:** 2018-07-24

**Authors:** Ervin Taso, Vladimir Stefanovic, Ivana Stevanovic, Danilo Vojvodic, Aleksandra Topic, Aleksandra Petkovic-Curcin, Kosovka Obradovic-Djuricic, Aleksa Markovic, Mirjana Djukic, Dragana Vujanovic

**Affiliations:** ^1^Clinic for Stomatology, Military Medical Academy, Belgrade, Serbia; ^2^Institute for Medical Research, Military Medical Academy, Belgrade, Serbia; ^3^Department for Biochemistry, Faculty of Pharmacy, University of Belgrade, Belgrade, Serbia; ^4^Department for Prosthetics, Faculty of Dental Medicine, University of Belgrade, Belgrade, Serbia; ^5^Department for Oral Surgery, Faculty of Dental Medicine, University of Belgrade, Belgrade, Serbia; ^6^Department for Toxicology, Faculty of Pharmacy, University of Belgrade, Belgrade, Serbia

## Abstract

Biocompatibility of dental materials (DM) can be evaluated by gingival crevicular fluid (GCF) oxidative stress (OS) status. The goal of the study was to ascertain influence of dental caries degree, teeth position, and type and amount of applied DM on GCF OS profile. For this purpose, we tested six DMs that were sealed in one session: amalgam (Amg), composites: Tetric EvoCeram and Beautifil (BF), phosphate cement—zinc phosphate and polycarboxylate cements—zinc polycarboxylate cements, and glass ionomer cement (GIC). The study included 88 dental outpatients. Follow-up was scheduled at 7th and 30th day. Oxidative stress parameters (malondialdehyde (MDA) and glutathione (GSH) levels and total superoxide dismutase (tSOD) activity) were measured before (0th day) and after the treatment (7th and 30th day) in GCF. Control teeth were mirror-positioned healthy teeth. The DM accomplished the following effects (listed in descending order): increase of GSH in GCF was realized by ZPoC > BF > GIC > Amg; tSOD activity increase by ZPoC > BF > Amg; and MDA decrease by ZPoC > ZPhC > Amg > TEC. Dental caries provokes insignificant rise of OS in GCF. ZPoC and ZPhC showed the highest antioxidant effect, contrary to GIC. Restorations with antioxidant properties may reduce gum diseases initiated by caries lesion, what is of great clinical relevance in dentistry.

## 1. Introduction

Convincing evidence concerning oxidative stress- (OS-) associated dental pathologies (parodontopathy, oral cavity cancer, etc.) has been reported during recent decades [1–3]. Up-to-date studies on redox status in oral environment have referred mainly to peroxidase activity in saliva [2, 4–7]. Reports in 2017 turned researchers' attention to gingival crevicular fluid (GCF) as a diagnostic tool for oral diseases analysis and treatment outcome. The impact of oral environmental stressors (hygienic food and eating habits, smoking, etc.) on saliva is much more intense than on GCF, though it was reported that smoking instantly increases GCF flow [8, 9].

Leading by the fact that GCF is a very specific oral cavity fluid (a transudate of blood plasma placed in the gingival sulcus), less exposed to oral environmental stressors compared to saliva, which requires noninvasive sampling, we chose GCF as an appropriate oral matrix for this kind of testing [8]. Herein, we tested the influence of dental caries (a bacterial disease of the dental hard tissues, also defined as a final stage of local teeth immune response to oral pathogen invasion) as well as six dental fillings on GCF redox homeostasis [10].

Recently, it was documented that cell redox activity, that is, antioxidant defense against environmental stressors (including smoking, i.e., nicotine) has important implications on periodontal disease and is important [9]. It is well acknowledged that OS (or other type of stress) is an inability of antioxidative defense system in living organisms to cope with free radicals (FRs) overproduction that results in oxidative injury of all classes of biomolecules, including proteins, lipids, phospholipids, and deoxyribonucleic acid. Different classes of FRs (reactive oxygen, nitrogen, sulfur, or carbon species (ROS, RNS, RSC, or RCC)) can initiate corresponding type of stress, oxidative, nitrosative, thiyl, or carbonyl stress (OS, NS, TS, and CS), respectively [11]. Along with changed cell signalization and energy breakdown, the overall occurrences finally end up with a cell death, by apoptosis [12].

Overproduction of ROS (including superoxide anion (O_2_^•−^), hydrogen peroxide (H_2_O_2_), hydroxyl radical (HO^•^), and hypochlorous acid (HOCl)) occurs in dental lesions (caries) during phagocytosis. Reactive species injure subcellular and/or cellular membranes of phagolysosomes and/or neutrophils during respiratory burst. Over time, oxidation products of polyunsaturated fatty acids (cell membrane ingredients) become converted into carbonyls, such as malondialdehyde (MDA), a reliable marker of lipid peroxidation (LPO) [13]. Together with myeloperoxidase and NADH-oxidase, they leak out of phagolysosomes into phagocyte cytosol and further at a site of infection or inflammation and damage phagocytes and injure tissue. Reports on exogenously present myeloperoxidase assume that it enhances bacterial phagocytosis and intracellular killing by macrophages.

Accordingly, total superoxide dismutase (SOD) (covers cytosolic and extracellular form (Cu/Zn-SOD) and mitochondrial (Mn-SOD), as well) converts O_2_^•−^ into H_2_O_2_, which further becomes converted into H_2_O, by catalase. These biochemical reactions can attenuate myeloperoxidase-induced bactericidal activity within or out of phagocytes and reduce myeloperoxidase-associated lipid peroxidation (LPO) [11, 14]. The role of SOD in dental pathologies has not been investigated until now.

In support of the possible redox interactions of the tested dental restoratives is the fact that some xenobiotics undergo redox metabolism and contribute to O_2_^•−^ production [15]. Hitherto, testing of dental materials' pro or antioxidant activity has not been implemented in biocompatibility type of analysis *in vitro* and *in vivo.*

By measuring GSH, MDA, and tSOD in GCF, we studied its redox response to dental caries and six dental restorations, considering the dental lesion degree, teeth position, and placed amount into teeth.

## 2. Material and Methods

The study was carried out on dental outpatient from the Clinic for Stomatology at the Military Medical Academy, Belgrade, Serbia, for 30 days, in accordance with the International Ethical Guidelines and Declaration of Helsinki (1964/1975) and was approved by the Ethical Committee of the Military Medical Academy, Ministry of Defense, Serbia (Preference number VMA/10-12/A.1). The participants were introduced with the essence of the study and planned procedures, filled out a questionnaire dental record form related to general and oral health, and gave written consent to participate in this study.

### 2.1. Patients

The 88 dental outpatients, aged 18–70, were recruited by the tabular specified criteria (Table 1).

In respect to Black's Classification Criteria, patients were classified into four groups (K2–K5), and according to the type of the applied dental fillings, patients were divided into six groups: Amg, TEC, BF, ZPhC, ZPoC, and GIC. Position of the treated teeth were also presented (Table 2) [16, 17].

### 2.2. GCF Sampling Procedure

The GCF sampling was performed by the filter paper technique. After removing supragingival biofilm, the sampling area (with sterile cotton rolls) was gently air dried 1 min before the sampling procedure. A paper strip (Perio-paper, Pro Flow, Amityville, NY, USA) was inserted into the gingival/periodontal sulcus/pocket until mild resistance and left for 30 seconds [18]. Strips contaminated with blood or saliva were discarded. The volume of taken GCF was measured by Periotron 6000 (Interstate Drug Exchange, Amityville, NY, USA), previously calibrated. The GCF sampling paper strips were placed into microcentrifuge plastic tubes. Elution of GCF was performed with 500 *μ*L phosphate-buffered saline by vortexing for 10 seconds and centrifugation at 3000 g for 5 min, to remove plaque and cellular elements. The supernatants were transferred into Eppendorfs and stored at −70°C until OS analysis.

**Scheme 1:**
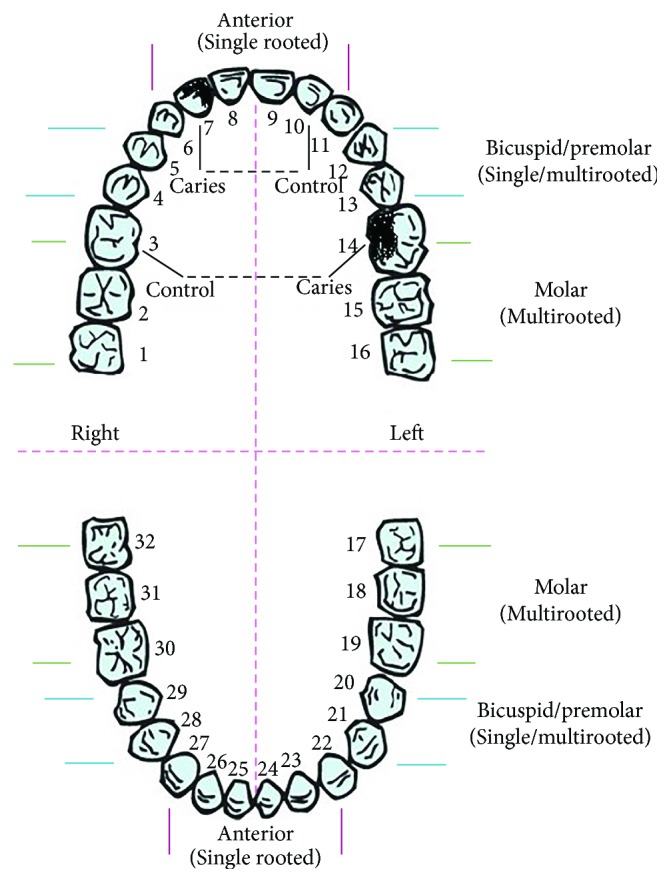
Teeth diagram. Scheme taken form https://www.pinterest.com/pin/290763719669177256/ is modified by the illustration of teeth with caries and corresponding controls.

The sampling of GCF adjacent to treated teeth was performed three times (prior and two times after the treatment (0th, 7th, and 30th day)), while GCF sampling from the corresponding healthy mirror-positioned, that is, antagonistic healthy teeth was done, once, at 0th day (Scheme 1).

### 2.3. Dental Restorations

Dental fillings (temporary and permanent) were sealed in one session, and placed mass refers to the range 0.07–2.03 g (Table 3).

Used dental fillings referred to temporary restorations: ZPhC (Cegal NV, Galenika, R Serbia) and ZPoC (Harvard, USA); permanent restorations: Amg (Extracap D caps, Galenika, R Serbia); and two nanohybrid composites that require UV light for binding in cavity: BF (the mixture of bisphenol-A-diglycidyl-dimethacrylate (BisGMA) 15–25%, triethylene-glycol-dimethacrylate (TEGDMA) 12–14%, aluminofluoro-borosilicate glass 50–60%, aluminium trioxide (Al_2_O_3_) 1-2%, and DL-Camphorquinone) (Shofu, Japan)) and TEC (the mixture of 2.5–10% of BisGMA and 2.5–10% of urethane-dimethacrylate (UEDMA) and nonhazardous additions (Ivoclar Vivadent, USA)); GIC (GC Fuji PLUS*®,* Green Circle, USA) was used for both settings, stand-alone restorations and the base for nanohybrid composites (BF and TEC).

### 2.4. Measurement of Oxidative Stress Markers in GCF

#### 2.4.1. Malondialdehyde Measurements

Malondialdehyde, LPO biomarker was measured spectrophotometrically by thiobarbituric acid reactive substances (TBARS) production method. In brief, MDA forms red-colored compound with TBA reagent (15% TCA and 0.375% TBA, water solution, pH 3.5) during the incubation at 95°C, measured at 532 nm. Data were expressed as nmol MDA/mg proteins [19].

#### 2.4.2. Superoxide Dismutase Measurements

Superoxide dismutase (EC 1.15.1.1.; SOD) activity was measured spectrophotometrically, as an inhibition of epinephrine oxidation to colored product adrenochrome by O_2_^•−^. Kinetics of SOD activity was measured at 480 nm after the addition of 10 mmol epinephrine into samples prepared in carbonate buffer (50 mmol, pH 10.2), containing 0.1 mmol EDTA [20]. Data were expressed as U SOD/mg proteins.

#### 2.4.3. Glutathione Measurements

The reduced form of glutathione (GSH) reduces Elman's reagent [5,5′-dithiobis (2-nitrobenzoic acid), DTNB] (36.9 mg DTNB in 10 ml of methanol) in TRIS-HCl buffer (0.4 M, pH −8.9) into yellow colored 5-thio-2-nitrobenzoic acid (TNB) [21]. Produced TNB is proportional to the amount of depleted GSH (on the account of its oxidation) and was determined spectrophotometrically (at 412 nm), by the enzymatic recycling assay. The results were expressed as nmol TNB/mg proteins.

### 2.5. Protein Measurements

Total protein concentrations were estimated in supernatants of GCF samples according to Lowry et al. method [22].

### 2.6. Statistical Analysis

The appropriate statistical analysis for this type of results after determining the normality of data distribution is the analysis of covariance ANCOVA, since we compare teeth with the corresponding control. The ANOVA test is inappropriate since it excludes the individuality (the corresponding matches for single patient) and implies overall values.

In more details, the one-sample Kolmogorov-Smirnov normality test followed by nonparametric Wilcoxon signed-rank test for two related samples and two-tailed independent *t*-test were used to analyze the differences between OS parameters in GCF adjacent to control healthy teeth (healthy teeth mirror positioned) and untreated teeth with caries (K2–K5, 0th day). The impact of six applied restorations on the OS parameters was tested when data were analyzed in respect to both independent variables, degree of caries (K groups) and/or type of applied restorations, 2 × 2 between-group analysis of covariance (ANCOVA), and post hoc comparisons (least-significant difference (LSD)) were used.

The influence of filling mass on OS parameter was estimated by nonparametric Spearman's correlation analysis, while association between teeth position and filling mass was analyzed by Pearson correlation 2-tailed test.

In all performed analyses, dependent variables were OS parameters in GCF from 7th to 30th day, while those on 0th day were used as a covariate to control individual differences in therapy outcome (A-set of analyses). Value *p* ≤ 0.05 was considered statistically significant.

Two statistical programs SPSS 17.0 were used for the above analyses and Excel Microsoft program, version 2016, for graphical data presentation.

## 3. Results

Since we did not have enough patients within some of the formed groups (referring to the degree of dental caries—groups K2-K5, and the applied restorations—6 groups: Amg, TEC, BF, ZPhC, ZPoC, and GIC), we cross-examined GCF OS status before and after the applied treatments.

**Figure 1:**
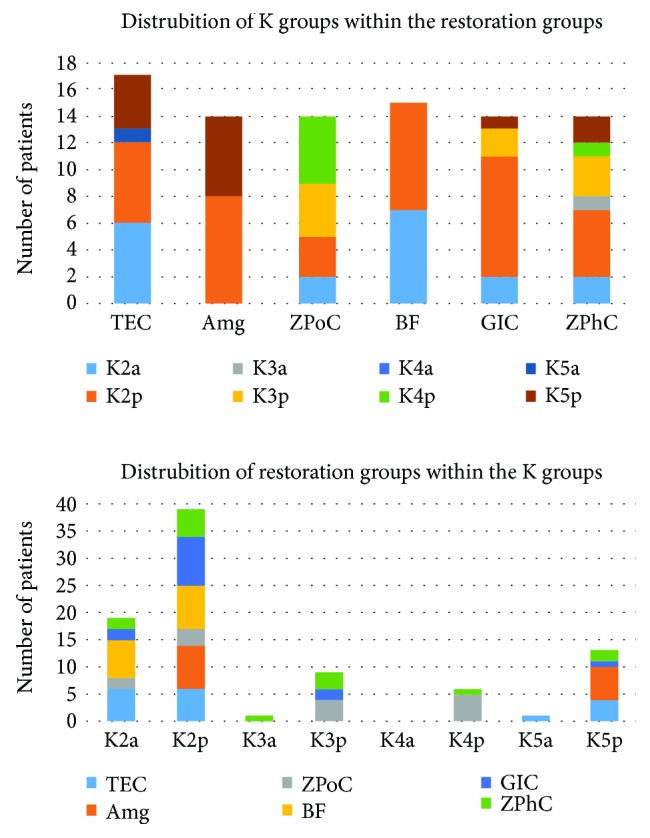
Distribution of patients across the groups obtained on the basis of two criteria: K2–K5 groups and six restoration groups. The representation of certain groups within the groups obtained on the basis of the other criteria. Suffixes a and p in the K groups' nomenclature indicate teeth position, anterior and posterior, respectively. Data corresponds to the data given in Table 2.

The number of patients treated with certain dental fillings within the K groups and opposite were presented in Figure 1. Percentages of that distribution (extracted from Table 2) were mentioned in descending order, where is appropriate, within this section.

Multiple estimation approaches were performed to test the influence of caries (four K categories) and restorations (six types of dental fillings) on OS status (tSOD, GSH, and TBARS) in GCF.

**Figure 2:**
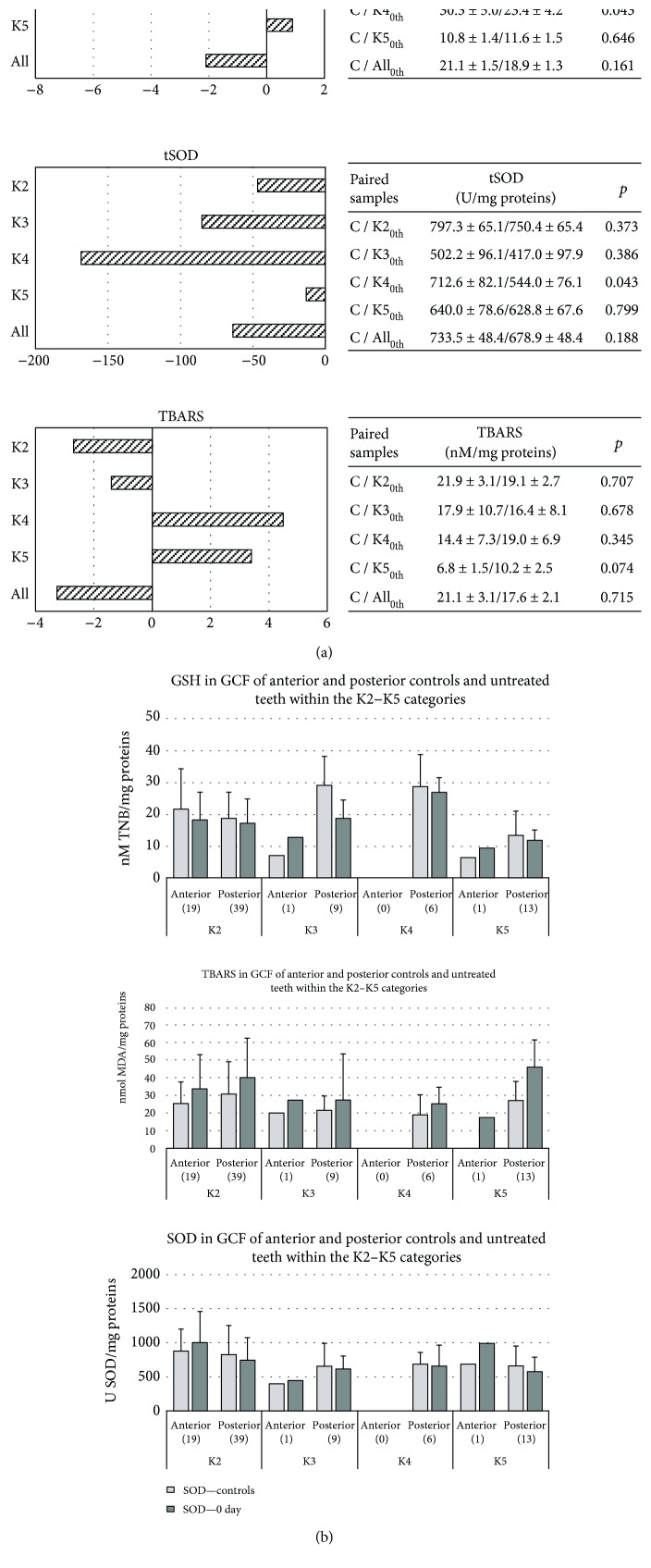
(a) The impact of caries degree on OS parameters. Differences in OS markers in GCF between controls and untreated teeth with caries (0th day) in respect to Black's classification (K2–K5): GSH was expressed as nmol TNB/mg proteins; LPO, that is, TBARS as nmol MDA/mg proteins and tSOD activity as U SOD/mg proteins. Zero line represents mean of the controls. Tables on the right show mean ± standard error of OS parameters obtained in two related samples and differences between them (p) from all patients. The number of patients within the K groups (0th day) was as follows: K2–58, K3–10, K4–6, and K5–14 (in Table 2). Nonparametric Wilcoxon signed-rank test for two related samples was used. *p* ≤ 0.05 value was considered statistically significant. (b) GCF redox status in anterior and posterior controls and pretreated teeth within the K2–K5 categories. Groups K2–K follow the Black's Classification. Teeth position: separated posterior and anterior teeth. GSH was expressed as nmol TNB/mg proteins; LPO, that is, TBARS as nmol MDA/mg proteins and tSOD activity as U SOD/mg proteins. Controls: corresponding antagonistic “mirror”- positioned teeth; 0th day: pretreated teeth with caries.

Initially, we determined differences of OS markers within the healthy controls (to reveal if teeth position affects GCF OS status) and then compared pretreated teeth (0th day) with corresponding health control teeth (to test if caries by itself affects redox status in tooth decay degree dependent manner) (Figure 2(a)). No significance was observed, except that GSH and tSOD activities were lower (*p* = 0.043, in both cases) within K4 group, compared to control teeth. Data were presented as histograms in Figure 2(b).

**Figure 3:**
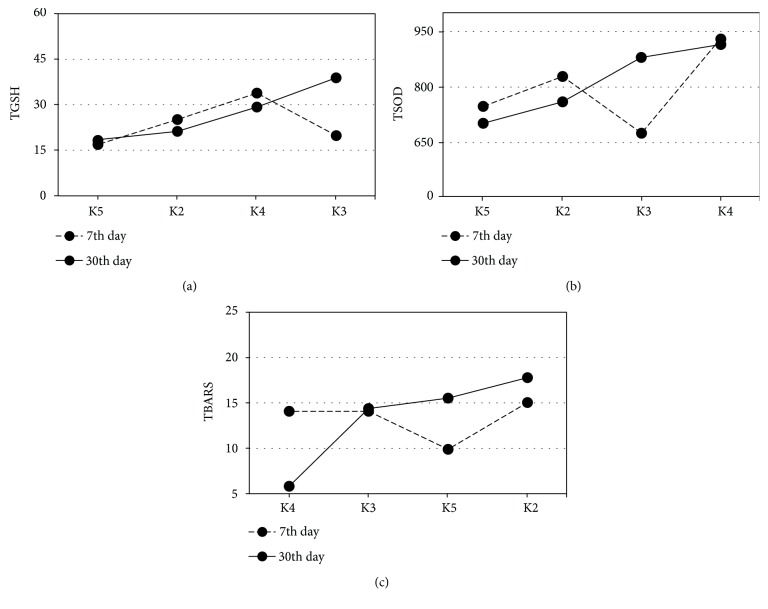
GCF redox status of pre- and posttreatment period within the K2–K5 groups. Estimated marginal means for OS parameters at 7th and 30th day were evaluated with 0th day, in regard to Black's classification (K2–K5): (a) GSH covariates at the 0th day was 18.4 nmol TNB/mg proteins; significant difference was found in 30th day between K2-K3 (*p* = 0.001) and K3–K5 (*p* = 0.001); (b) tSOD covariate at the 0th day was 675.8 U SOD/mg protein; (c) TBARS covariate at the 0th day was 18.1 nmol MDA/mg proteins; significant difference was found in 30th day between K2 and K4 (*p* = 0.026). The patients' distribution across the 4 K groups is tabulated (Table 2). 7th and 30th days were presented with a dash and solid line, respectively. 2 × 2 between-group analysis of covariance (ANCOVA) and post hoc comparisons (least-significant difference (LSD)) was used. *p* ≤ 0.05 value was considered statistically significant.

**Figure 4:**
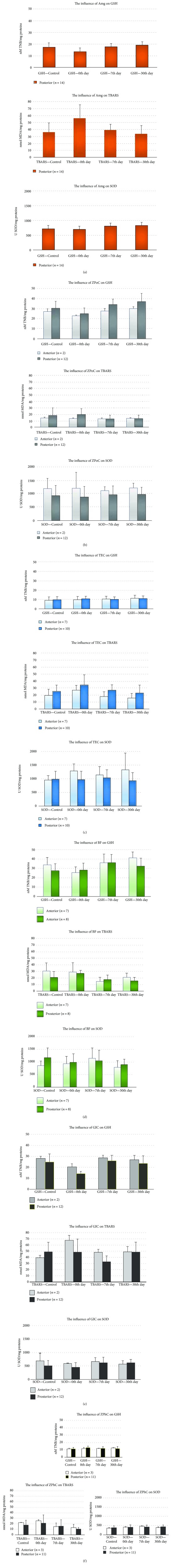
(a–f) The influence of tested restorations on GCF redox status of dental patients. OS parameters (GSH, MDA, and SOD) in GCF were presented in respect to teeth position (anterior and posterior) with given number of patients per posterior and anterior treated teeth. (a) The influence of Amg on GCF redox status of dental patients. Amg group: patients with posterior treated teeth only (*n* = 14 patients): 8 were from K2 and 6 from K5 group. The amount of sealed Amg: 1.318571 ± 0.71267 g. (b) The influence of ZPoC on GCF redox status of dental patients. Patients with posterior and anterior treated teeth (*n* = 14 patients): 12 patients with posterior treated teeth (3 from K2, 4 from K3, and 5 form K4 group) and 2 patients with anterior treated teeth (2 from K2 group). The amount of sealed ZPoC for anterior was 0.035 ± 0.025 g and posterior was 0.229 ± 0.167 g. (c) The influence of Tetric EvoCeram on GCF redox status of dental patients. Patients with posterior and anterior treated teeth (*n* = 17 patients): 10 patients with posterior treated teeth (6 from K2 and 4 from K5 group) and 7 patients with anterior treated teeth (6 from K2 and 1 from K5 group). The amount of sealed TEC for anterior was 0.029 ± 0.02253 g and posterior was 0.152 ± 0.159 g. (d) The influence of BF on GCF redox status of dental patients. Patients with posterior and anterior treated teeth (*n* = 15 patients): 8 patients with posterior and 7 with anterior treated teeth (all from K2 group). The amount of sealed BF for anterior: 0.029 ± 0.014 g and posterior 0.064 ± 0.042 g. (e) The influence of GIC on GCF redox status of dental patients. Patients with posterior and anterior treated teeth (*n* = 14 patients): 12 patients with posterior treated teeth (9 from K2, 2 from K3, and 1 from K5 group) and 2 patients with anterior treated teeth, from K2 group. The amount of sealed GIC for anterior was 0.035 ± 0.015 g and posterior was 0.168 ± 0.153 g. (f) The influence of ZPhC on GCF redox status of dental patients. Patients with posterior and anterior treated teeth (*n* = 14 patients): 11 patients with posterior treated teeth (5 from K2, 3 from K3, 1from K4, and 2 from K5 group) and 3 patients with anterior treated teeth (2 from K2 and 1 from K3 group). The amount of sealed ZPhC for anterior was 0.08 ± 0.081 g and posterior was 0.235 ± 0.145 g.

GCF OS status of pre- (0th day) and posttreatment period (7th and 30th day) within K2–K5 groups was presented in Figures 3 and 4(a)–4(f). The highest GSH and tSOD activities were documented in the K3 group, at 30th day: (K3: ZPhC 40%, ZPoC 40%, and GIC 20%); GSH was significantly higher in K3 than in K2 (^∗∗^*p* = 0.001) and K5 (^∗∗^*p* = 0.001), at 30th day) (K2: BF 26%, TEC 21%, GIC 19%, Amg 14%, ZPhC 12%, and ZPoC 8% and K5: Amg 43%, TEC 36%, ZPhC 14%, and GIC 7%). The lowest MDA was obtained in K4 group (K4 group: ZPoC 83% and ZPhC 17%) on 30th day, and it was significantly lower compared to K2 (*p* = 0.026), at 30th day for MDA (Figure 3).

Data were presented as histograms in Figures 4(a)–4(f).

**Figure 5:**
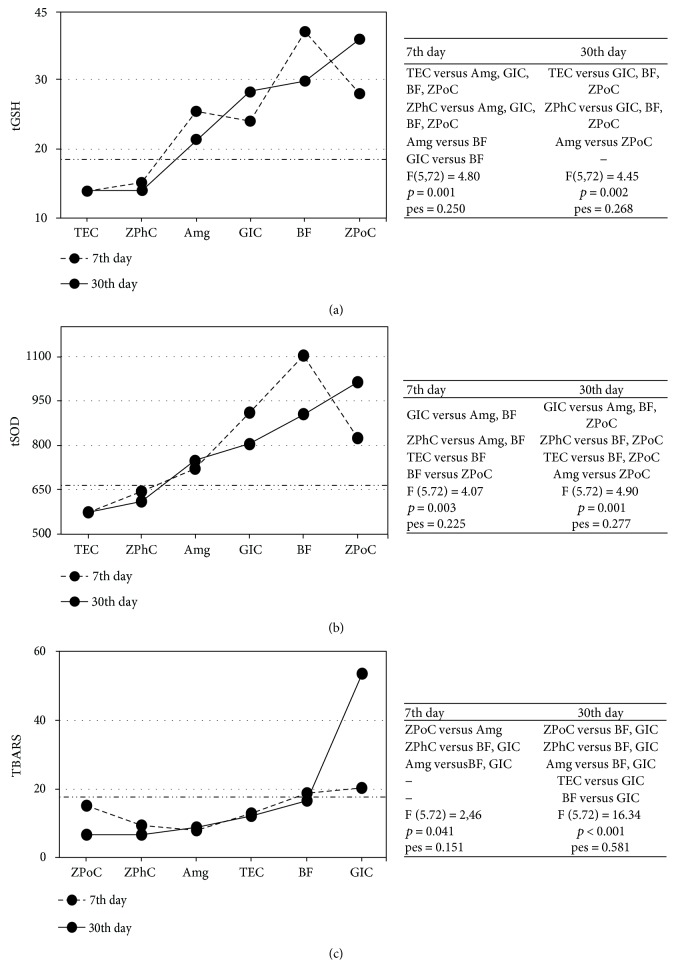
The influence of the restorations on OS parameters before and after the treatments. Estimated marginal means for OS parameters in GCF at 7th and 30th day were evaluated with 0th day (horizontal line: long dash dot dot). In regard to the applied restorative, (a) GSH covariate at the 0th day was 19.3 nmol TNB/mg proteins; (b) tSOD covariate at the 0th day was 665.6 U/mg proteins; (c) TBARS covariate at the 0th day was 17.8 nmol MDA/mg proteins. The patients' distribution across the K groups and restorative groups is tabulated (Table 2). 7th and 30th days were presented with a dash and solid line, respectively. Tables on the right show differences (*p* values) in OS parameters between restoratives' treatment groups. pes: partial eta squared. 2 × 2 between-group analysis of covariance (ANCOVA) and post hoc comparisons (least-significant difference, (LSD)) was used. *p* ≤ 0.05 value was considered statistically significant.

Significant beneficial influence of the applied restorations on the certain OS markers in GCF mainly occurred at 30th day and are listed in descending order: elevated GSH was obtained by ZPoC > BF > GIC > Amg and tSOD activity by ZPoC > BF > Amg; while decreased MDA was gained by ZPoC > ZPhC > Amg > TEC (Figure 5).

Higher tSOD activity was accomplished in anterior, compared with posterior teeth, on 30th day (*p* = 0.018).

No association was confirmed for filling mass and OS parameters. Significant correlation was obtained between filling mass and teeth position (Table 3) (Pearson correlation: 0.307, *p* = 0.004).

## 4. Discussion

Current reports on OS-associated dental/periodontal pathologies have mainly been related to peroxidase activity in saliva. Redox profile differs across oral environmental compartments including hard dental tissue, saliva, and GCF [1, 23]. Herein, we tested the influence of dental caries and six dental fillings on GCF OS homeostasis, which recently has been recognized as reliable diagnostic fluids for periodontal diseases and drug analysis [8].

Hence, physiology of GCF depends on teeth position (anterior includes incisors and canines versus posterior includes premolars and molars), size, shape, root characteristics, function related to pressure at bite, and so on; herein, we compared OS status of GCF across controls and teeth with caries, before (0th day) and after the treatments (7th and 30th day) individually, for each patients, by using ANCOVA statistics [24, 25]. Adhering to the inclusion criteria (that also cover smokers that smoke less than one pack of cigarettes/day) (Table 1) and comparing individually the obtained results for the treated teeth with the control teeth (for each patient), the study was carefully designed to minimize bias.

We ascertained that OS status of GCF is not associated with teeth position, except that GSH was insignificantly elevated in posterior teeth, though we should recall that the posterior teeth prevailed over the anterior in our patients (Table 2, Figures 2(a) and (b)). Contrary to the reports of Davis et al., we showed insignificant OS development with dental degree, from K2–K4, but accordingly, we obtained slightly lower OS in K5 group, what was probably a consequence of reduced central blood supply and teeth metabolic processes, thus diminished local antioxidant defense [26]. According to the literature, we showed that the lowest GSH and tSOD activities were in K4 group (^∗^*p* = 0.043) [17, 19, 27]. Slightly higher GSH level in K5 group may be explained by reduced metabolic activities, due to insufficient blood supply (Figure 2(a)).

The reason of reduced tSOD activity in K4 group (^∗^*p* = 0.043) (Figure 2(a) and 2(b)) may be prescribed to the lack of the substrate, O_2_^•−^. Also, O_2_^•−^ reacts easily with nitrogen monoxide to form harmful peroxynitrite anion (this reaction is three times faster than dismutation catalyzed by SOD). This last mentioned reaction is involved in the acetylation of amino acids, accomplished by gram-negative anaerobes (*Porphyromonas gingivalis, Prevotella nigrescens,* etc.) [28]. In accordance with the literature, we showed slightly increase of LPO in advanced dental lesion, confirming OS development with caries progression (Figure 2(a)). This notion is supported by ROS overproduction via NADPH oxidase and myeloperoxidase during phagocytosis of bacterial pathogens and their interactions with two main targets in membrane phospholipids, double bond between C-atoms and the ester linkage between glycerol and fatty acids [1, 29, 30]. Stick to dental caries is a bacterial inflammation accompanying with local immune response [10]. Placed within lysosomes (the azurophilic granules of phagocytes) of neutrophils, NADPH oxidase and myeloperoxidase produce ROS during so-called “respiratory burst.” NADPH oxidase catalyzes superoxide anion (O_2_^•−^) production through a large oxygen (O_2_) consumption (when >80–90% of O_2_ becomes converted into O_2_^•−^), while myeloperoxidase catalyzes production of several reactive species, such as hypohalogenated acids (including hypochlorous acid (HOCl)) in reactions of hydrogen peroxide (H_2_O_2_) and halide ions (Cl^−^, Br^−^, and I^−^); hypothiocyanous acid (HOSCN) from H_2_O_2_ and halide and pseudohalide ions; hydroxyl radical (HO^•^), via non-Fenton reaction between O_2_^•−^ and HOCl; and nitrating intermediates, *in vivo* [31–34]. After being fused with lysosomes, phagosome (a vesicle formed around engulfed bacteria) matures into phagolysosomes, within the neutrophils. That is the point when intracellular killing of pathogens starts by ROS. Although ROS effects occur intracellularly, within phagolysosomes, they are diffusible and can react outside of phagolysosomes, within the neutrophils and surrounding tissues (for instance with GCF, in case of dental caries) [19]. The reactive species produced by myeloperoxidase are responsible for the oxidation, chlorination, and nitration of cytosolic proteins, glycoproteins, and lipoproteins in neutrophils or in nearby tissues (i.e., HOCl chlorinates amines and produces toxic chloramine, or HOSCN inhibits glycolysis and energy supply, etc.) and are responsible for the side effect of inflammation (death of phagocytes and tissue damage) [34–36].

Development of OS in GCF of teeth with caries was anticipated since immunoinflammatory-associated occurrences, such as caries, are characterized by ROS overproduction, depletion of reducing equivalent sources, such as NAD (P) H and GSH, and oxidative injure of biomolecules, including lipids (Figures 2(b)).

As to the effect of the restorations on OS profile of GCF, ANCOVA analysis of the data sorted by the Black's Classification Criteria (Figures 1 and 3) showed that the highest GSH and tSOD activities were documented in the K3 group (ZPhC = ZPoC > GIC), at 30th day, what was significant for GSH compared to K2 (BF > TEC > GIC > Amg > ZPhC > ZPoC) (^∗∗^*p* = 0.001) and K5 (Amg > TEC > ZPhC > GIC) (^∗∗^*p* = 0.001) and reduced LPO in K4 group (ZPoC> > ZPhC), what was significantly lower compared to K2 (^∗^*p* = 0.026), at 30th day for MDA. From this, we concluded that ZPoC and ZPhC, within the K3 group, have more (and equal) supportive role in increasing tSOD activity and GSH. To emphasize that, ZPoC notably reduced LPO within the K4 group.

Accordingly, ANCOVA analysis of the data arranged in respect to the applied restorations showed significant GSH increase by the following restorations listed in descending order: ZPoC > BF > GIC > Amg; and tSOD activity increase by ZPoC > BF > Amg; while MDA decrease was gained by ZPoC > ZPhC > Amg > TEC (Figures 4(a)–4(f) and 5). Consistent with the literature, we confirmed that ZPoC and ZPhC demonstrated profound antioxidant effect in comparison to the other used dental fillings, in terms of suppressed LPO and GSH regaining, contrary to GIC which demonstrated completely opposite, prooxidant effect, while composites, BF and TEC did not show noticeable effects on GCF OS status [37, 38].

According to the literature, the most profound antioxidant effect of ZPoC and ZPhC can be prescribed to hydrolysis of their acid components (itaconic and maleic acids versus phosphoric acid, resp.) [37, 39]. Dicarboxylic acids, such as itaconic and maleic acids, are used as monomers for biopolymers (resins or synthetic fibers). Lampropoulou et al. acclaimed itaconate as a major physiological regulator of the global metabolic rewiring and effector functions of inflammatory macrophages. It regulates succinate levels and function, mitochondrial respiration, and inflammatory cytokine production during macrophage activation [39]. Adhering to this, accomplished antioxidant role of ZPoC (especially in the suppression of LPO within GCF) probably comes from itaconic acid. On the other hand, phosphoric acid binds many divalent cations, including transient metals (iron, cooper, etc.). It is well known that transient metals (in low valent states) participate in Fenton reaction to produce the most potent ROS, HO^•^ (no enzymatic system exists in living organisms to scavenge it) [40]. It is used in dentistry as an etching, that is, corrosive solution. Corrosives kill pathogens and prevent locally bacterial diseases, including dental caries. The antioxidant effect of ZPhC was confirmed by all three OS markers.

From all applied restorations, only GIC accomplished prooxidant property (suppressed tSOD activity and elevated LPO). According to the literature, the explanation for such occurrences lies in fluoride anion (released from GIC) interactions with metal cations embedded in antioxidant metalloenzymes, such as SOD, catalase, and peroxidase. The obtained results are consistent with Yamaguti et al.'s study in which it was shown that low-dose fluoride treatment affects antioxidant enzymes, including SOD and catalase (CAT), and rises LPO in parotid and submandibular salivary glands of rats. Explicitly, they demonstrated that fluoride intoxication caused more pronounced OS in submandibular than in parotid salivary glands [38].

It is well known that prolonged leaching of small amount of unbound monomers (1.5–5%), such as TEGDMA for instance, is blamed for cytotoxic and other systemic effects of composites. The leaching of methacrylate monomers occurs because of the incomplete UV polymerizations of composites during sealing process [41]. Herein, the amount of the TEGDMA, present in the sealed composites (BF and TEC), was almost >300 times lower than its subtoxic dose (<4 mM), reported by Gul et al., thus adverse/toxic effects (including disruption of redox homeostasis in GCF) were completely avoided [41–43]. Individual sensitivity of the patients with polymorphism of GSH to TEGDMA molecule was reported [44]. Additionally, low GSH levels in GCF of dental patients treated with TEC contrary to BF may relate to monomer UEDMA [45].

The low levels of GSH, tSOD activity, and MDA measured in K5 group before and after the dental restoration strengthening depraved influence of insufficient blood supply and metabolism on GCF profile.

Positive correlation between filling mass (0.07–2.03 g) and teeth position (Pearson correlation: 0.307, *p* = 0.004) was anticipated concerning the size of the anterior and the posterior teeth.

## 5. Conclusion

Taking into consideration the influential factors such as dental lesion degree, type of applied dental fillings, and teeth position, we made the following conclusions: (i) GCF OS status does not depend on teeth position and does not differ between healthy teeth; (ii) untreated teeth with caries do not differ significantly from corresponding controls (exclusion: elevated GSH in posterior teeth); (iii) reduced GSH and MDA were recognized as a more reliable and sensitive OS marker than tSOD; (iv) ZPoC and ZPhC achieved profound antioxidant effect; (v) none of the applied restorations accomplished complete antioxidant effect, while GIC realized prooxidant effect; and (vi) restorations with antioxidant properties may reduce gum diseases initiated by caries lesion.

To our knowledge, this is the first paper on this topic and performed with dental patient. Restorations with antioxidant properties may reduce gum diseases initiated by caries lesion, what is of great clinical relevance in dentistry. We showed and recognized that redox interactions may influence dental material biocompatibility; thus, evaluation of GCF OS status may be considered as a useful tool in biocompatibility testing of dental fillings.

## Figures and Tables

**Table 1:** Patients' recruited criteria.

The inclusion criteria	Criteria related to teeth condition
	(i) The proximal caries on anterior and posterior teeth
	(ii) The existence of the same type of antagonistic teeth (“mirror”-positioned healthy teeth) used controls
	(iii) An absence of fresh postextractional or traumatic wounds in the restoration area or the area of restored surfaces
	(iv) An absence of infection in the area of restored surfaces
	Other influencing criteria
	(i) Patients with no bone-associated diseases or treatments
	(ii) Satisfactory oral hygiene
	(iii) Reliable and cooperative patients

The exclusion criteria	Criteria related to teeth condition
	(i) Endodontic and/or periodontal infections in the area of cervical filling
	(ii) Parodontopathy
	(iii) Prominent periodontal pockets
	(iv) Subgingival caries
	(v) Fillings that were prominent outside the cavity
	Other influencing criteria
	(i) Patients on radiation immunosuppressive therapy
	(ii) Patients bone-associated diseases and malignant diseases
	(iii) Addictive patients on drug/alcohol/cigarettes (>20 cigarettes per day)
	(iv) Bad oral hygiene
	(v) Unreliable and uncooperative patients

Inclusion criteria: criteria related to teeth condition. The proximal caries on the anterior and posterior teeth. The existence of the same type of antagonistic teeth (“mirror”-positioned healthy teeth) used controls. An absence of fresh postextractional or traumatic wounds in the restoration area or the area of restored surfaces. An absence of infection in the area of restored surfaces. Other influencing criteria. Patients with no bone-associated diseases or treatments. Satisfactory oral hygiene. Reliable and cooperative patients. Exclusion criteria: criteria related to teeth condition. Endodontic and/or periodontal infections in the area of cervical filling. Parodontopathy. Prominent periodontal pockets. Subgingival caries. Fillings that were prominent outside the cavity. Other influencing criteria. Patients on radiation immunosuppressive therapy. Patients' bone-associated diseases and malignant diseases. Addictive patients on drug/alcohol/cigarettes (>20 cigarettes per day). Bad oral hygiene. Unreliable and uncooperative patients.

**Table 2:** Distribution of patients according to Black's Classification Criteria with defined teeth position and applied type of restoratives.

*Dental restoratives*	*K2* *n* = 58	*K3* *n* = 10	*K4* *n* = 6	*K5* *n* = 14
*TEC*	12 (21) 6/6	—	—	5 (36) 4/1
*Amg*	8 (14) 8/0	—	—	6 (43) 6/0
*ZPoC*	5 (8) 3/2	4 (40) 4/0	5 (83) 5/0	—
*BF*	15 (26) 8/7	—	—	—
*GIC*	11 (19) 9/2	2 (20) 2/0	—	1 (7) 1/0
*ZPhC*	7 (12) 5/2	4 (40) 3/1	1 (17) 1/0	2 (14) 2/0

The number of patients within the K groups were given in respect to the type of applied restoration and in bracket (the percentage of the patients treated with the certain restoration in respect to all patients within the appropriate K group). Also, the number of patients with posterior/anterior positioned teeth was indicated (P/A).

**Table 3:** The amount of sealed restoratives in regard to teeth position.

*Type of restoration*	*Posterior*	*Anterior*
*Number of patients (P/A)*	*(Number of patients)*	*(Number of patients)*
***Amg, *** *n* = 14* (P/A 14/0)*	**1.32 ± 0.71**	/
	*n* = 14	
***ZPoC, *** *n* = 14* (P/A 12/2)*	**0.23 ± 0.167**	**0.04 ± 0.025**
	*n* = 12	*n* = 2
***TEC, *** *n* = 17* (P/A 10/7)*	**0.15 ± 0.159**	**0.03 ± 0.023**
	*n* = 10	*n* = 7
***BF, *** *n* = 15* (P/A 8/7)*	**0.06 ± 0.042**	**0.03 ± 0.014**
	*n* = 8	*n* = 7
***GIC, *** *n* = 14* (P/A 12/2)*	**0.17 ± 0.153**	**0.04 ± 0.015**
	*n* = 12	*n* = 2
***ZPhC, *** *n* = 14* (P/A 11/3)*	**0.24 ± 0.145**	**0.08 ± 0.081**
	*n* = 11	*n* = 3

## Data Availability

The data used to support the findings of this study are available from the corresponding author upon request.

## Abbreviations

Al_2_O_3_: Aluminium trioxide
Amg: Amalgam
BF: Beautifil
BisGMA: Bisphenol-A-diglycidyl-dimethacrylate
DTNB – 5: 5-Dithiobis (2-nitrobenzoic acid)
GCF: Gingival crevicular fluid
GIC: Glass ionomer cement
H_2_O_2_: Hydrogen peroxide
LPO: Lipid peroxidation
MDA: Malondialdehyde
NS: Nitrosative stress
O_2_^•−^: Superoxide anion
OS: Oxidative nitrosative stress
TBARS: Thiobarbituric acid reactive substances
TEC: Tetric EvoCeram
TEGDMA: Triethylene-glycol-dimethacrylate
TNB: 5-Thio-2-nitrobenzoic acid
GSH: Glutathione
tSOD: Total superoxide dismutase
UEDMA: Urethane-dimethacrylate
ZPhC: Zinc phosphate cement
ZPoC: Zinc polycarboxylate cement.
